# Non-anemic Iron Deficiency from Birth to Weaning Does Not Impair Growth or Memory in Piglets

**DOI:** 10.3389/fnbeh.2016.00112

**Published:** 2016-06-14

**Authors:** Alexandra Antonides, Serana van Laarhoven, Franz J. van der Staay, Rebecca E. Nordquist

**Affiliations:** ^1^Behaviour and Welfare Group (Formerly Emotion and Cognition Group), Department of Farm Animal Health, Faculty of Veterinary Medicine, Utrecht UniversityUtrecht, Netherlands; ^2^Brain Center Rudolf Magnus, Utrecht UniversityUtrecht, Netherlands; ^3^Adaptation Physiology Group, Institute of Animal Sciences, Wageningen UniversityWageningen, Netherlands

**Keywords:** iron deficiency, cognition, development, spatial learning, memory, pigs, anemia

## Abstract

Early iron deficiency is associated with impaired (cognitive) development, the severity of which depends on the timing and duration of the under-supply of iron. To design effective treatment and prevention strategies for iron deficiency in humans, suited animal models are needed. In an earlier study (Antonides et al., 2015b) we separated 10 pairs of piglets from their mothers within a few days after birth and reared one sibling with artificial iron-deficient (ID) and the other with balanced control milk until weaning. ID piglets grew slower and showed poorer reference memory (RM) performance than their controls in a spatial holeboard task, even weeks after iron repletion. One putative intervening factor in that study was pre-weaning maternal deprivation. In an attempt to refine the piglet iron-deficiency model, we assessed whether piglets reared by sows, but withheld iron supplementation, can serve as animal model of iron deficiency. As sow milk is inherently ID, piglets normally receive a prophylactic iron injection. Ten pairs of piglets were housed with foster sows until weaning (4 weeks). One sibling per pair was randomly assigned to the control group (receiving iron dextran injections: 40 mg iron per kilogram body mass on days 3 and 10), the other to the ID group. From weaning, all pigs were fed a balanced commercial diet. Blood samples were taken in week 1, 3.5, 6, and 12. Pre-weaning blood iron values of ID piglets were lower than those of controls, but recovered to normal values after weaning. Hemoglobin of ID piglets did not reach anemic values. Hematocrit and hemoglobin of ID animals did not decrease, and serum iron even increased pre-weaning, suggesting that the piglets had access to an external source of iron, e.g., spilled feed or feces of the foster sows. Growth, and spatial memory assessed in the holeboard from 10 to 16 weeks of age, was unaffected in ID pigs. We conclude that sow-raised piglets are not a suitable model for iron-deficiency induced cognitive deficits in humans. Based on our previous and the present study, we conclude that growth and memory are only impaired in piglets that suffered from pre-weaning anemia.

## Introduction

Iron deficiency is a form of malnutrition that is caused by a nutritional shortage of the micronutrient iron. The need for iron increases considerably during growth, pregnancy, and lactation (McLean et al., 2009; Gambling et al., 2011). If iron shortage during early development is not restored quickly enough, neonates are at risk of developing iron deficiency, and eventually anemia. Suckling neonates run a great risk of developing iron deficiency because of the low iron supplies in maternal milk (Fransson and Lönnerdal, 1980; Bates and Prentice, 1994). Worldwide, ~2 billion people suffer from iron deficiency, of which the highest prevalence is among children under the age of five (McLean et al., 2009). Early-life iron deficiency in humans is known to cause retarded growth and irreversible deficits in the development of motor and cognitive skills and memory functions (Beard, 2003; Lozoff and Georgieff, 2006). The timing and duration of the under-supply of iron is crucial for the severity of these adverse effects. Rodent models have elucidated some important mechanisms involved in the development of iron deficiency, on both the (neuro)physiological and behavioral level. Results from both human and rodent studies suggest that early-life iron deficiency causes irreversible deficits in brain structure and function (Yehuda and Youdim, 1989; Lozoff and Georgieff, 2006).

In addition to rodents, pigs have recently received increasing attention as animal model species in translational research (e.g., Lind et al., 2007; Kobayashi et al., 2012). Although widely used, rodent models for human conditions have recently raised concerns. The reliability of these small animal models is questioned, as successful pre-clinical studies in rodents scarcely translate to effective clinical use in humans (van der Worp et al., 2010; Macleod, 2011). Large animal models may show less discrepancies in study outcomes with humans. Animals that more closely resemble humans are likely to yield more reliable and thus more relevant study results (Festing and Altman, 2002). In this light, the pig is thought to be a more suited and promising animal model species for translational research than rodents (Gieling et al., 2011). In addition, pigs have a (neuro)anatomy, physiology, and developmental pattern that more closely resemble that of humans (Conrad et al., 2012). As in humans, the pig's brain growth spurt occurs perinatally, whereas in rodents it occurs postnatally (Dobbing and Sands, 1979).

The pig in particular seems to be a suited animal model to study the effects of early-life iron deficiency on development (Miller and Ullrey, 1987). Piglets are born with low iron supplies of ~50 mg, which is mostly found in hemoglobin (Venn et al., 1947). Their rapid growth causes a need for 7–10 mg iron daily in the first weeks of life (Venn et al., 1947; Svoboda and Drabek, 2005). Sow milk provides piglets with only around 1 mg of iron per day, which does not suffice to provide the piglets with the amount of iron needed (Csapó et al., 1996). It is believed that piglets of the wild boar, the ancestor of the pig, ingest iron through rooting in soil. In the pig farming industry, however, the barren environment in which piglets are reared provides no such external source of iron. Moreover, for decades pig breeders have been selecting pigs for increasingly larger litter sizes and fast growth (Rauw et al., 1998). This may increase the need for iron and cause more severe iron deficiency in neonatal piglets (Furugouri and Kawabata, 1975).

In pig husbandry, it is therefore common practice to provide new-born piglets with an iron dextran injection, to prevent development of iron deficiency anemia. Recently, we conducted a study to investigate the effects of dietary induced pre-weaning iron deficiency in piglets on spatial learning and memory (Antonides et al., 2015b). We used 10 pairs of piglets from 10 different litters. One piglet from each pair was randomly assigned to the iron deficiency group, the other served as control. The control piglets were administered an iron injection; the iron deficient (ID) treatment animals received a saline injection. Animals were then separated from the sow at 4 to 6 days of age, and fed artificial milk diets for 28 days. Control animals received a balanced milk diet, whereas ID animals received an iron deficient milk diet. After treatment, all piglets were weaned and fed a balanced piglet diet. ID piglets showed impaired RM learning capability in a spatial holeboard task (measured after iron repletion) as well as impaired growth (permanent), lower blood iron values (during treatment) and lower brain iron concentrations (measured 8 weeks after iron repletion). ID animals were clinically anemic at the end of treatment, as assessed by their hemoglobin values.

In a similar study by Rytych et al. (2012), piglets were assigned to a control, mildly ID or severely ID diet from 2 to 28 days of age, during which their growth, blood parameters and cognitive performance was assessed. Control animals received an iron injection. Severely ID piglets showed impaired learning in a double T-maze task, whereas mildly ID piglets showed deficits in reversal learning. All ID animals (mild and severe) became anemic. Treatment did not affect growth of the piglets. This lack of effect may have been due to the short duration of the study (28 days).

However, the piglets in both studies (Rytych et al., 2012; Antonides et al., 2015b) were separated from the sow within a few days after birth, in order to feed them the controlled experimental diets. This is very early compared to separation from the sow in industrial pig husbandry, where piglets are usually weaned at around 4 weeks of age. Early-life maternal deprivation is known to increase long-term stress responses in rats (Suchecki and Tufik, 1997) and to cause intellectual damage in human infants (Yarrow, 1961). Rat pups that experienced 24 h of maternal separation at postnatal day 9 showed reduced hippocampal plasticity at adulthood (Roceri et al., 2002). This shows that such a stressful and emotional event during early brain development may cause permanent deficits in brain function. The hippocampus is involved in spatial learning and memory (Bird and Burgess, 2008) and hippocampal dysfunctions are known to impair spatial learning and memory (Olton et al., 1978; Da Silva et al., 1986).

Complementing this finding, maternal deprived rats showed delayed acquisition and a higher degree of persistent behavior in the Morris water maze task compared to control animals (Oitzl et al., 2000). As the holeboard is also a spatial learning and memory task, these results raise the concern whether the results from our previous iron deficiency study (Antonides et al., 2015b) may have been influenced by maternal deprivation. Oitzl and colleagues showed that maternal deprivation in their rats amplified individual behavioral differences at senescence. The lasting detrimental effects of iron deficiency on memory performance found in our previous study might also have been influenced or amplified by early maternal deprivation. In order to refine the piglet iron deficiency model, early maternal deprivation should ideally be prevented in the experimental set-up. In addition, this would increase the welfare of the piglets, as they are spared the experience of separation stress at an extremely young age.

In the present study, we investigated whether piglets that stayed with the sow until weaning without receiving iron supplementation might serve as a suited, refined animal model for iron deficiency in humans. To this end, the piglets were allowed to consume only sow milk (neither iron supplementation nor additional feed were provided) until weaning at 4 weeks of age.

We measured hematology, growth, and spatial memory performance in ID and control piglets. Based on previous findings of pre-weaning, dietary induced iron deficiency in piglets (Rytych et al., 2012; Antonides et al., 2015b), we expected that ID piglets would develop anemia as indicated by their hemoglobin values. Also, pre-weaning hemoglobin, hematocrit, and serum iron values were expected to be lower in ID animals than in control animals. We predicted that ID piglets would show retarded growth and impaired spatial orientation performance, indicated by lower spatial memory scores in the cognitive holeboard task. The results of this study may yield a refined, more natural and less invasive piglet model of iron deficiency.

## Materials and methods

### Ethics note

This study was reviewed and approved by the local animal ethics committee of Utrecht University (DEC, DierExperimenten Commissie) and was conducted in accordance with the recommendations of the EU directive 86/609/EEC. All efforts were made to minimize the number of animals used and to avoid suffering.

### Animals and housing (pre-weaning)

Pairs of piglets [(Terra × Finnish landrace) × Duroc] from 12 different litters, all born within the same week, were selected from the commercial pig breeding farm of the Faculty of Veterinary Medicine, Utrecht University, The Netherlands (see Supplementary Table 1 for an overview of the data of selected piglets and foster sows). All piglets of each litter were weighed within 24 h after birth, including stillborn piglets. The pair of piglets of the same sex that had a weight closest to the average weight of the litter was selected and marked with a differently colored ear tag. For two sibling pairs, due to poor growth and reduced health of one of the siblings, a sibling of a different gender was selected, resulting in two male-female sibling pairs. One of the piglets of each selected pair of piglets was assigned randomly to the control group, the other to the ID group.

All piglets remained with their maternal sow during the first 2 days after birth, to allow them to ingest colostrum. Then, the selected piglets were transferred to one of three foster sows at 2 days of age and were exchanged for piglets of the foster sow. We ensured that these foster sows had more teats available than the number of piglets to nourish, to avoid teat competition and increase survival chances. The three foster sows fed 9, 11, and 12 piglets. The use of foster sows was chosen in order to only withhold additional feed from the piglets selected for our study, leaving all other piglets of the piggery unaffected by our experiment. Otherwise, we would have had to withhold all piglets of the 12 selected litters additional feed. This would have reduced their growth performance and thus have caused substantial financial losses for the piggery. Cross-fostering piglets at a young age is done on a regular basis in conventional practice, for example to increase survival chances of low birth weight piglets (Deen and Bilkei, 2004). Heim et al. (2012) showed that early postnatal cross-fostering does not affect behavior, survival, or growth performance of adopted piglets.

All selected piglets remained with the foster sows until weaning at 4 weeks of age. During this period, they did not receive any additional milk replacer or creep feed and thus only had access to sow milk, which is low in iron content (Csapó et al., 1996). Water was available through a drinking nipple. During the first 4 weeks (pre-weaning) all selected piglets were weighed once every 2 days in order to closely monitor their growth. One pair of piglets was excluded from the experiment before weaning due to poor growth, which necessitated our interference with extra care and additional feed. Thus, the experiment was conducted with 11 sibling pairs, consisting of 12 male and 10 female piglets. After weaning, all piglets were weighed once per week until they had reached the age of 17 weeks, which was when the experiment ended.

### Iron treatment and blood collection

On day 3 and day 10 after birth, control animals received an iron dextran injection of 0.2 ml/kg body weight, containing 200 mg Fe/ml (MS FerroPig, Schippers Export B.V., The Netherlands), as adapted from Lipiñski et al. (2010). This corresponded to 40 mg Fe/kg body weight, based on the weight on the day of injection. We decided to adjust the injections to body weight and not to birth weight (as in Lipiñski et al., 2010) as this, we argue, is more accurate. The ID piglets received two saline injections of 0.2 ml/kg body weight. Lipiñski and colleagues argue that the amount of iron normally administered in conventional practice (100–200 mg per animal) is excessive and may even be toxic. They showed that reducing and spreading the iron administration reduces iron toxicity and allows the body to use the iron more effectively. In the present study, we therefore used this administration scheme.

At the age of 1, 3.5, 6, and 12 weeks, blood samples were taken from the jugular vein. Piglets were fixated by hand during all blood sampling moments except at 12 weeks of age, when a pig snare was used to briefly restrain the animals. Blood hemoglobin and hematocrit were determined using the Siemens ADVIA® 2120i System with ADVIA Multispecies Testing software. Serum iron was determined using the Beckman Coulter UniCel DxC 600 according to standard procedure.

### Experimental housing (post-weaning)

At ~4 weeks of age, all selected piglets were weaned and transported to the experimental facility (located next to the farm they originated from), where they were housed in two adjacent pens (both 4 × 5 m) from November 2014 until February 2015. Sibling pairs were separated and housed in different pens. Growth of the piglets was expected to be more balanced if piglets in a pen were of a more uniform weight (Francis et al., 1996), therefore in forming the groups, pigs of similar weights were housed together. Both groups contained a balanced number of ID and control animals (see Supplementary Table 1). Each piglet received a sprayed number on its back to allow fast identification. At the experimental facility, all piglets received commercial pig feed *ad libitum* (Prevent piglet feed, De Heus Voeders BV, Ede, The Netherlands). Feed was provided in a large feeding trough in order to reduce feeding competition (4 × 0.30 m). Water was available *ad libitum* through a drinking nipple.

The pens had a concrete floor covered with straw as bedding material. Each pen contained a wooden nest box (3 × 1.5 m) with plastic flaps along the front for easy access and heat preservation. Inside the nest box, two heat lamps hang ~1 m above the floor, which were removed when the piglets were 12 weeks old. Nest box flooring consisted of rubber mats and a thick layer of sawdust and straw as bedding. Different sized plastic balls and pig sticks were provided as enrichment. The stable in which the pens were located was naturally lighted and ventilated (not heated). Lights were on from 7:30 a.m. until 4:30 p.m. Temperature ranged from −1 to 14°C and was recorded daily. After the end of the study, all pigs were fattened to slaughter weight and transported to and slaughtered in a commercial slaughter house.

### Holeboard apparatus

The spatial cognitive holeboard for pigs (built by Ossendrijver BV, Achterveld, The Netherlands) was a square arena measuring 5.30 × 5.30 m, with gray synthetic 80 cm high walls with a steel bar on top. The square testing arena was surrounded by a 40 cm wide corridor and contained a guillotine door on each side through which piglets could enter the arena (for details, see Antonides et al., 2015a). The apparatus had a blue plastic slatted floor. The arena contained 16 possibly rewarded sites in a four by four matrix, consisting of plastic food bowls (Road Refresher Large, Prestige Pet Products, Essex, England). These bowls contained false bottoms; in the actual bottoms of all bowls, three M&M's® were placed daily in order to assure that the piglets could not use olfactory cues to find the rewards. To prevent piglets from using visual cues to find the rewards, each food bowl was covered with a red plastic ball (JollyBall Dog Toy, Ø24 cm, 1400 g, Jolly Pets, Ohio, USA). The piglets always entered the corridor clockwise and were allowed to leave the arena through the door closest to the main entrance of the apparatus. During testing, pen mates were housed in the waiting pen in front of the holeboard apparatus. This allowed the piglet inside the arena to smell and hear, but not see its pen mates during testing. It also served as an auditory extra-maze cue for the piglet during testing. The fluorescent lights on the sloping ceiling of the stable and the position of the experimenter standing in the corridor of the holeboard arena served as visual extra-maze cues for spatial orientation within the arena. During testing, eye contact with the animal that was tested was avoided.

The experiment was controlled through a laptop which collected the data. A sensor in the food bowl sent a signal whenever the connection with the magnet in the ball was lost, which was the case when the ball was lifted by the piglet with its snout. This signal was registered via an interface (LabJack), transferred to the laptop and processed by a custom-made software program (Blinq Systems, Delft, The Netherlands). If a ball was lifted multiple times within a 10-s period and no other food bowl was visited in between, this series of events was counted as one single visit. The entry door was randomly determined by the software before each trial and was opened manually by the experimenter before a piglet entered the corridor, using a rope and pulley system. It has been demonstrated in a study using rats as subjects that randomization of the start position forestalled the development a preferred pattern of visiting the holes (van der Staay et al., 1990). This effect of randomizing start positions has since then been corroborated in a number of holeboard studies (reviewed in van der Staay et al., 2012).

### Habituation and holeboard training

During the first week after arrival, piglets were allowed to get accustomed to their new pen and pen mates. During this week, they were habituated to humans for 30–60 min per day. Afterwards, all pigs were gradually habituated to the hallway leading to the holeboard apparatus and its waiting area. Piglets were habituated to the holeboard arena from 6 to 10 weeks of age. At the start of these holeboard habituation sessions, all piglets of a pen were allowed to enter the holeboard arena and corridor together, with all arena doors left open. During these sessions, all food bowls contained multiple food rewards. The balls were lifted to facilitate the learning process of finding food rewards, and bowls were refilled as soon as the rewards in a bowl were consumed. The group size of pigs let into the holeboard together was gradually decreased over the habituation sessions, until piglets were comfortable to enter the arena alone. The balls were gradually lowered to allow piglets to learn to lift the balls to uncover the food bowls and find the rewards.

### Holeboard testing

When all piglets were physically able to lift the balls and were comfortable entering the arena alone, holeboard testing started. This was the case at 10 weeks of age. Each piglet received six habituation trials in which all 16 holes contained a reward; two trials per day on three successive days. After the habituation trials, the acquisition trials started, in which each piglet was assigned to its own configuration of four baited holes. In total, four different configurations were used in such a way that, across all piglets, every hole was baited equally often. Piglets received two daily trials in close succession (massed trials) on working days. If after at least 40 acquisition trials a predetermined learning criterion was reached (an average reference memory score of > 0.7 over the last four trials), the pigs were switched to the reversal configuration. The reversal configuration was the 180° rotated pattern of baited holes used during the acquisition phase. After maximally 60 acquisition trials, all piglets were switched to the reversal phase, regardless of their performance. All piglets received a total of 20 reversal trials.

### Holeboard variables

A trial was started manually when a piglet entered the arena with both front legs. A trial was ended automatically by the software when all rewards were found, or after 300 s, whichever event occurred first. For the six habituation trials preceding the acquisition phase (all holes baited), the total number of visits (TV) and the total number of rewards found (REW) were automatically recorded by the software. For the acquisition and reversal trials, the following variables (van der Staay et al., 2012) were either recorded or calculated automatically by the software:

- Working memory (WM), a ratio defined by the number of visits that yield a food reward divided by the number of visits and re-visits to the rewarded set of holes;
- Reference memory (RM), a ratio defined by the number of visits and re-visits to the rewarded set of holes divided by the number of visits and re-visits to all holes;
- Trial duration (TD) in seconds, the time between entering the holeboard and finding all four rewards, or the maximum TD of 300 s if the pig did not find all rewards;
- Inter-visit interval (IVI) in seconds, the average time between successive hole visits;
- Latency to the first visit (LFV) and Latency to the first rewarded visit (LFR) in seconds;
- Total visits (TV), unrewarded visits (URV) and rewarded visits (RV);
- Number of visits until the 1st (Vfirst), 2nd (Vsecond), 3rd (Vthird), and 4th (Vfourth) reward were found (for a detailed explanation see Gieling, 2013, pp. 173–176).

### Statistical analyses

Data were analyzed with the statistical software SAS (version 9.4, SAS Institute, Cary, NC, USA). Normal distribution of the residuals of all variables was assessed using the Shapiro-Wilk test (SAS PROC UNIVARIATE). Birth weights of animals used in the experiment were compared using a mixed model ANOVA with Litter as random effect. The effects of treatment on the growth curves from 4 to 17 weeks of age and on blood iron values were analyzed with a mixed model ANOVA to account for clustering of piglets within litters and repeated measurements within piglets, with the fixed effects Treatment (control or ID), Week, and their interaction, with a random effect for Litter. In case of significant interaction effects of Treatment by Week on the blood iron values, we additionally performed analyses on the separate blood sampling time points, in order to assess at which time points differences were found. For these individual analyses, a Bonferroni correction for multiple comparisons was applied. Note that the number of observations differed per blood collection moment and per variable due to technical difficulties during either blood collection or the analyses of the samples. For the number of observations per time point and per variable see Supplementary Table 2.

The six successive habituation trials were analyzed separately with a mixed model ANOVA with the fixed effects Treatment (control or ID), Trials and their interaction, with a random effect for Litter. For the acquisition and reversal phase, means of trial blocks (four successive trials each) were calculated for all variables. The first ten trial blocks of the acquisition phase (block 1-10) were analyzed; thus excluding the extra acquisition trials that a piglet received when it had not yet reached the preset criterion of RM > 0.7 after 40 trials. The reversal phase consisted of five trial blocks (block 11–15). All variables expressing latencies or durations were log_10_-transformed to meet the normality assumption. Two outliers in the data of the variable LFV were detected using the online outlier detector QuickCalcs (GraphPad Software, Inc., La Jolla CA, USA) and replaced by missing values. The effects of treatment on holeboard performance were analyzed with a mixed model ANOVA with the fixed effects Treatment (control or ID), Trial blocks and their interaction, with a random effect for Litter.

The holeboard data analyses were performed for three different phases: acquisition, transition, and reversal. The transition phase is the switch from the acquisition phase to the reversal phase, i.e., the last trial block of the acquisition phase compared to the first trial block of the reversal phase (block 10 compared to block 11). This is a measure of the response flexibility of an animal: a large difference means that the animal faced difficulties to adapt to the new situation.

## Results

### Blood iron values

**Figure 1:**
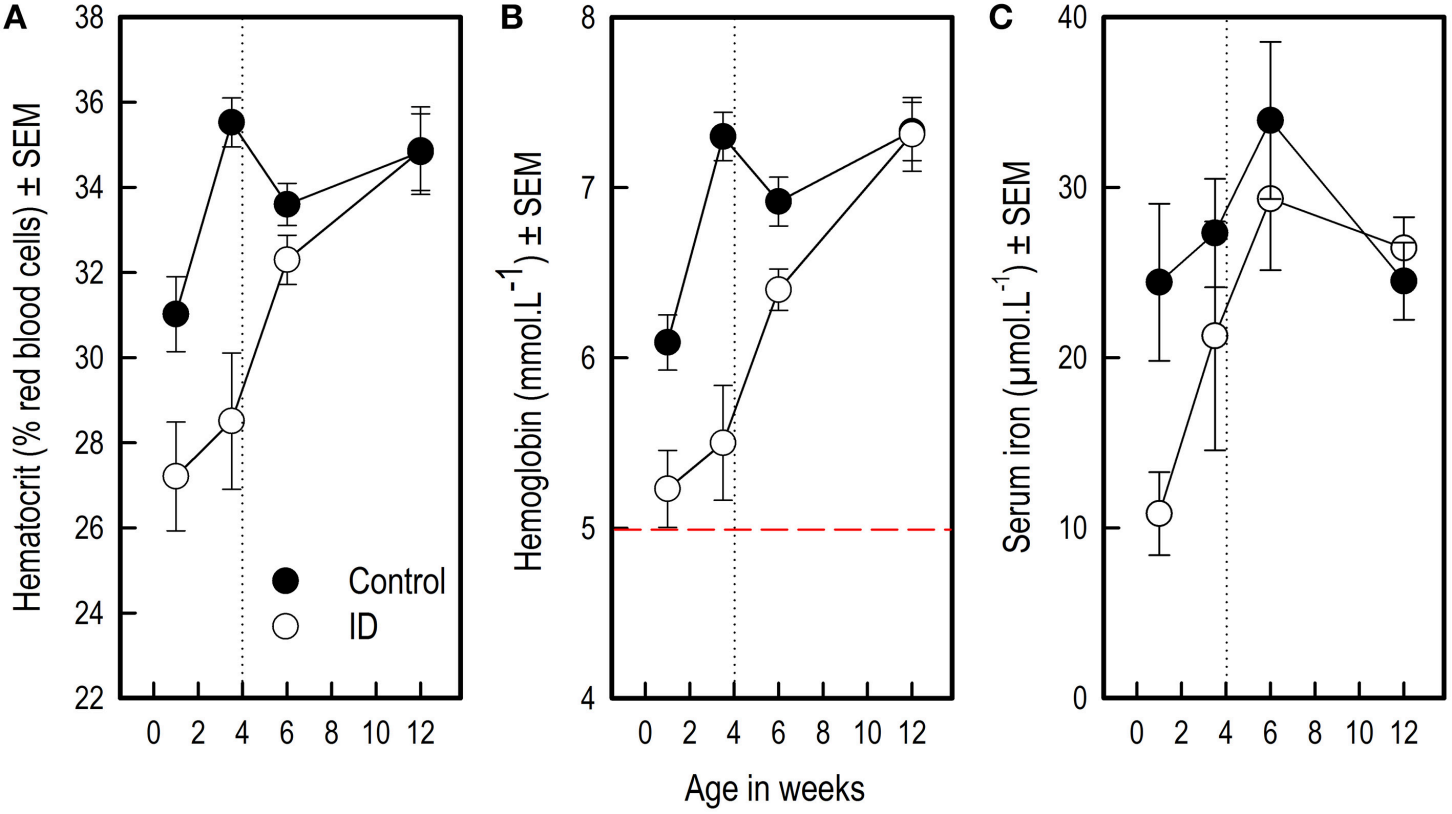
**Blood hematocrit, hemoglobin and serum iron values of ID and control animals from 1 to 12 weeks of age**. Weaning and transition to regular feed was at 4 weeks of age (dotted line). **(A)** Hematocrit values; **(B)** Hemoglobin values. The dashed red line indicates a hemoglobin value of 5, below which piglets are considered anemic (Ishaya, 2012); **(C)** Serum iron values. The number of successfully collected and analyzed samples varied per measure and sampling time point; for the number of successful observations per time point and measure, see Supplementary Table 2. Mean values and standard error of the mean (SEM) are depicted per treatment group and time point.

Hematocrit (Hct) and hemoglobin (Hb) values of ID animals were lower than those of control animals over the different sampling time points from 1 to 12 weeks of age (Treatment: both *p* < 0.0001, see Figure 1; Table 1). All blood iron values increased over time for all animals (Week: Hct and Hb: *p* < 0.0001; serum iron: *p* = 0.007). Both Hct and Hb showed a steeper increase in ID animals than in control animals (Treatment by Week interaction: Hct: *p* = 0.007; Hb: *p* = 0.001). Serum iron values of ID animals tended to show a steeper increase than those of control animals (Treatment by Week interaction: *p* = 0.05; Figure 1C).

**Table 1:** **Effects of feeding piglets iron-deficient sow milk as only nourishment until weaning on blood iron values**.

**Blood parameter**	**Treatment**			**Week**			**Treatment × Week**		
	***F***	***Df***	***P ≤***	***F***	***df***	***P ≤***	***F***	***df***	***P ≤***
**TREATMENT EFFECTS ON BLOOD PARAMETERS**									
**Hematocrit**	16.50	1.64	**<0.0001**	10.89	3.64	**<0.0001**	4.39	3.64	**0.0072**
**Hemoglobin**	20.87	1.64	**<0.0001**	26.88	3.64	**<0.0001**	6.04	3.64	**0.0011**
**Serum iron**	2.96	1.66	0.0900	4.37	3.66	**0.0072**	2.74	3.66	0.0503

*Blood parameters of ID and control animals are compared over the course of the experiment (0 to 12 weeks). Effects per sampling time point are listed in Table 2. Results that are considered significant (p < 0.05) are depicted in bold*.

#### Blood iron levels in ID animals during treatment

Visual inspection of Figure 1 suggests that blood iron values of ID animals rose between week 1 and week 3.5. Therefore, we compared blood iron values within ID animals between these sampling time points using one-sample t-statistics on the difference scores. These tests revealed that there was no difference in ID piglets' Hct (*t*_9_ = 0.95; *p* = 0.37) and Hb (*t*_9_ = 0.99; *p* = 0.35) values between week 1 and 3.5, whereas their serum iron values were higher in week 3.5 than in week 1 (*t*_9_ = 3.29; *p* = 0.01). Note that serum iron values were log_10_ transformed for this analysis to meet the normality assumption.

In order to investigate at which sampling time points treatment effects were present, we additionally analyzed differences between treatment groups at each separate sampling time point (Table 2). Because a Bonferroni correction was applied, differences with an associated *p*-value of < 0.01 were considered significant in these analyses.

**Table 2:** **Effects of feeding piglets iron-deficient sow milk as only nourishment until weaning on blood iron values per sampling time point in ID and control animals during the pre-weaning period (0–4 weeks) and after transition to regular feed (at 4 weeks of age; dotted line) are listed**.

**Age**	**Hematocrit**			**Hemoglobin**			**Serum iron**		
***wk***	***F***	***df***	***P ≤***	***F***	***df***	***P ≤***	***F***	***df***	***P ≤***
**EFFECTS OF TREATMENT PER SAMPLING TIME POINT**									
1	6.33	1.64	0.0144	10.58	1.64	**0.0018**	7.96	1.66	**0.0063**
3.5	17.38	1.64	**<0.0001**	25.16	1.64	**<0.0001**	0.62	1.66	0.4333
6	2.78	1.64	0.1002	6.95	1.64	0.0105	0.55	1.66	0.4590
12	0.00	1.64	0.9742	0.00	1.64	0.9863	0.47	1.66	0.4938

*Note that a Bonferroni correction was applied: effects of the treatment are considered statistically significant if p < 0.01 (depicted in bold)*.

#### Hematocrit

At 1 week of age, a measuring time point that fell between the two iron or saline injections, Hct values tended to be lower in ID animals than in control animals (*p* = 0.01, after a Bonferroni correction, this result was a trend). At the end of treatment and before weaning at 3.5 weeks, Hct values were lower in ID animals than in control animals (*p* < 0.0001). At 6 and 12 weeks of age, no differences in Hct values were found between treatment groups (Figure 1A; Table 2).

#### Hemoglobin

Hb values were lower in ID animals than in control animals at 1 week of age (*p* = 0.002) and at the end of treatment before weaning at 3.5 weeks of age (*p* < 0.0001). At 6 weeks of age, Hb values still tended to be lower in ID animals than in control animals (*p* = 0.01, after a Bonferroni correction, this result was a trend). No difference in Hb values between treatment groups were found at 12 weeks of age (Figure 1B; Table 2).

#### Serum iron

Serum iron values were lower in ID animals than in control animals at 1 week of age (*p* = 0.006). Near the end of treatment at 3.5 weeks and at 6 and 12 weeks of age, no differences in serum iron values between treatment groups were found (Figure 1C; Table 2).

### Weights and growth

**Figure 2:**
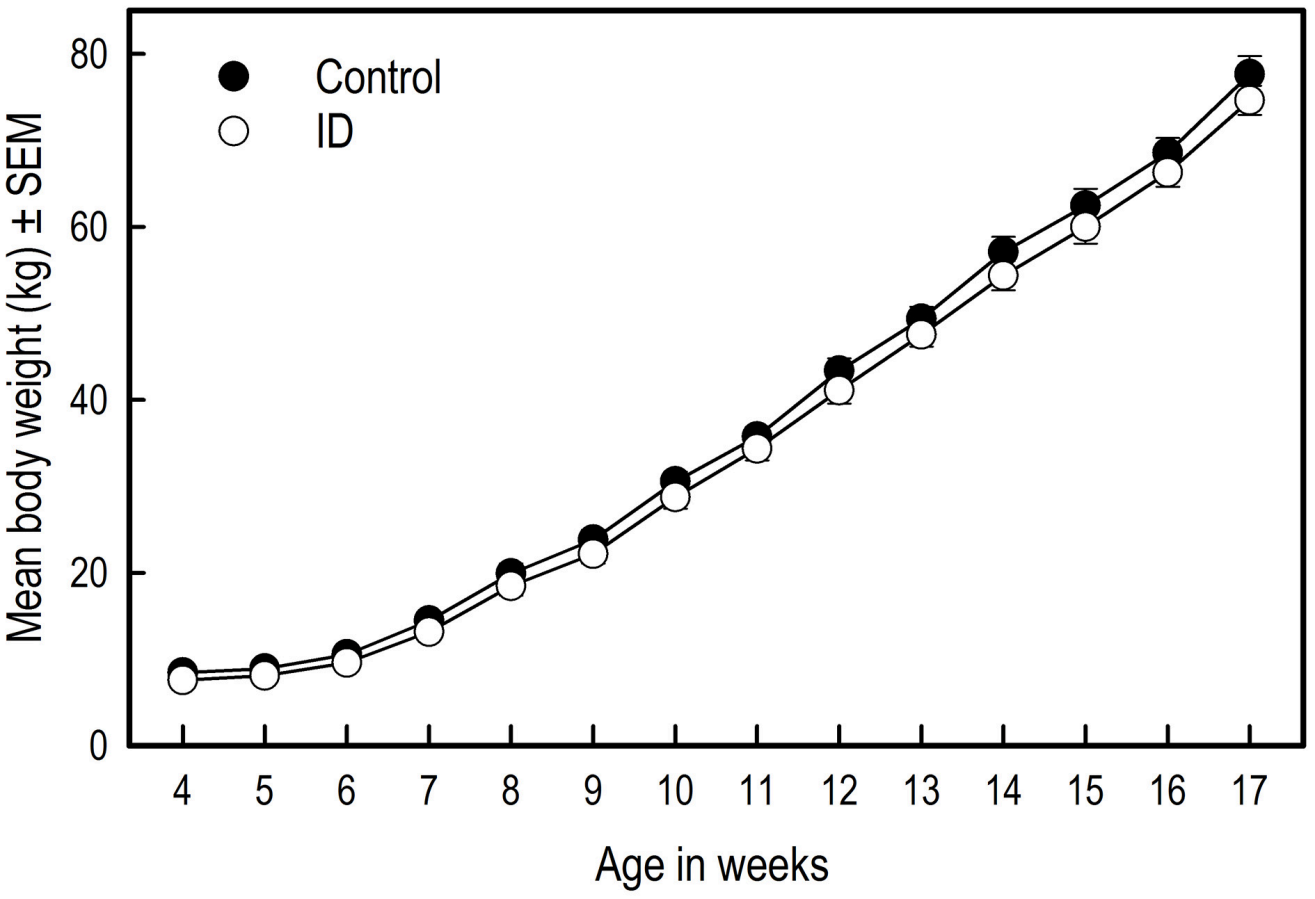
**Growth of ID and control piglets from weaning at 4 weeks of age to 17 weeks of age**. Mean values and standard error of the mean (SEM) are depicted per treatment group.

There was no difference in birth weight between siblings [*F*_(1, 10)_ = 2.22; *p* = 0.17]. Over the course of the experiment, there was no difference in average weight between treatment groups [Treatment: *F*_(1, 270)_ = 1.39; *p* = 0.24; Figure 2]. Weight gain in the two groups was similar [Week: *F*_(13, 270)_ = 395.79; *p* < 0.0001; Treatment by Week interaction: *F*_(13, 270)_ = 0.43; *p* = 0.96].

### Holeboard performance

The results of the statistical analyses of the holeboard data are listed in Table 3.

**Table 3:** **Effects of feeding piglets iron-deficient sow milk as only nourishment until weaning on performance in the spatial cognitive holeboard task during (A) The habituation phase (Hab) in which all 16 holes were baited, and (B) The acquisition phase (Acq), transition phase (Trans) and reversal phase (Rev)**.

**Measure**	**Phase**	**Treatment**			**Trials**			**Treatment** × **Trials**		
		***F***	***df***	***P ≤***	***F***	***Df***	***P ≤***	***F***	***df***	***P ≤***
**(A) HOLEBOARD HABITUATION (HAB)**										
Total number of visits (TV)	Hab	1.46	1.110	0.2302	0.56	5.110	0.7273	1.16	5.110	0.3349
Number of rewards found (REW)	Hab	1.28	1.110	0.2607	2.78	5.110	**0.0212**	0.72	5.110	0.6121
**(B) HOLEBOARD ACQUISITION (ACQ), TRANSITION (TRANS), REVERSAL (REV)**										
Working memory (WM)	Acq	0.22	1.190	0.6379	8.63	9.190	**<0.0001**	0.45	9.190	0.9034
	Trans	2.97	1.25	0.0969	25.32	1.25	**<0.0001**	0.75	1.25	0.3949
	Rev	0.01	1.74	0.9356	5.57	4.74	**0.0006**	0.77	4.74	0.5486
Reference memory (RM)	Acq	0.92	1.190	0.3390	50.60	9.190	**<0.0001**	0.42	9.190	0.9225
	Trans	0.00	1.27	0.9725	201.94	1.27	**<0.0001**	0.41	1.27	0.5269
	Rev	1.24	1.76	0.2687	38.19	4.76	**<0.0001**	1.22	4.76	0.3074
Trial duration (TD)	Acq	0.00	1.190	0.9904	15.54	9.190	**<0.0001**	0.79	9.190	0.6299
	Trans	1.06	1.27	0.3124	270.76	1.27	**<0.0001**	0.03	1.27	0.8531
	Rev	2.78	1.76	0.0998	27.61	4.76	**<0.0001**	0.88	4.76	0.4805
Inter-visit-interval (IVI)	Acq	0.00	1.190	0.9513	2.89	9.190	**0.0031**	0.66	9.190	0.7471
	Trans	0.53	1.27	0.7192	44.46	1.27	**<0.0001**	0.13	1.27	0.4728
	Rev	2.00	1.76	0.1617	10.01	4.76	**<0.0001**	0.88	4.76	0.4792
Latency first visit (LFV)	Acq	0.03	1.188	0.8584	4.11	9.188	**<0.0001**	0.91	9.188	0.5211
	Trans	0.04	1.27	0.8482	4.66	1.27	**0.0399**	0.75	1.27	0.3928
	Rev	0.18	1.76	0.6705	2.90	4.76	**0.0275**	2.54	4.76	**0.0464**
Latency first rewarded visit (LFR)	Acq	0.02	1.190	0.8796	6.66	9.190	**<0.0001**	1.12	9.190	0.3519
	Trans	1.08	1.25	0.3082	29.15	1.25	**<0.0001**	0.00	1.25	0.9611
	Rev	4.70	1.74	0.0334	9.50	4.74	**<0.0001**	0.05	4.74	0.9944
Total number of visits (TV)	Acq	0.03	1.190	0.8703	32.43	9.190	**<0.0001**	0.65	9.190	0.7548
	Trans	1.70	1.27	0.2028	52.36	1.27	**<0.0001**	1.36	1.27	0.2532
	Rev	0.17	1.76	0.6796	9.35	4.76	**<0.0001**	1.49	4.76	0.2133
Unrewarded visits (URV)	Acq	0.01	1.190	0.9063	42.87	9.190	**<0.0001**	0.70	9.190	0.7082
	Trans	1.23	1.27	0.2769	130.11	1.27	**<0.0001**	1.40	1.27	0.2464
	Rev	0.00	1.76	0.9829	17.15	4.76	**<0.0001**	1.71	4.76	0.1558
Rewarded visits (RV)	Acq	0.70	1.190	0.4050	1.73	9.190	0.0844	0.49	9.190	0.8791
	Trans	3.43	1.27	0.0750	7.16	1.27	**0.0125**	1.25	1.27	0.2735
	Rev	2.13	1.76	0.1488	4.21	4.76	**0.0039**	1.00	4.76	0.4139
Visits before 1st reward (Vfirst)^*^	Acq	0.60	1.190	0.4385	27.04	9.190	**<0.0001**	1.37	9.190	0.2046
	Trans	0.27	1.26	0.6080	34.85	1.26	**<0.0001**	0.18	1.26	0.6743
	Rev	0.28	1.75	0.6000	12.35	4.75	**<0.0001**	1.95	4.75	0.1103
Visits before 2nd reward (Vsecond)^*^	Acq	0.12	1.190	0.7342	28.03	9.190	**<0.0001**	1.13	9.190	0.3431
	Trans	0.09	1.24	0.7701	74.79	1.24	**<0.0001**	0.14	1.24	0.7072
	Rev	0.02	1.72	0.8774	18.09	4.72	**<0.0001**	1.24	4.72	0.3034
Visits before 3rd reward (Vthird)^*^	Acq	0.70	1.188	0.4048	39.33	9.188	**<0.0001**	0.67	9.188	0.7372
	Trans	0.00	1.22	0.9908	90.91	1.22	**<0.0001**	0.02	1.22	0.8940
	Rev	0.02	1.68	0.9026	18.61	4.68	**<0.0001**	0.57	4.68	0.6869
Visits before 4th reward (Vfourth)^*^	Acq	0.03	1.184	0.8525	34.04	9.184	**<0.0001**	0.68	9.184	0.7225
	Trans	7.99	1.19	**0.0108**	232.98	1.19	**<0.0001**	6.82	1.19	**0.0171**
	Rev	0.07	1.61	0.7934	30.92	4.61	**<0.0001**	4.81	4.61	**0.0019**

*The transition phase is the switch from the acquisition phase to the reversal phase, i.e., the last trial block of the acquisition phase compared to the first trial block of the reversal phase. Results that are considered significant (p < 0.05) are depicted in bold*.

* *For further information about the operational definitions of these variables, see (Gieling, 2013)*.

In the six habituation trials in which all 16 holes contained rewards, there was no treatment effect on the total number of visits made (TV) nor on the number of rewards found (REW), which in this phase is also an indication of the number of different holes visited. For all animals, TV did not change throughout these six trials. However, REW increased for all animals during this phase (Trials: *p* = 0.02), i.e., the piglets learned to visit more of the 16 food bowls during a trial. There was no Treatment by Trial interaction for TV or REW (Table 3A).

#### Working memory and reference memory

**Figure 3:**
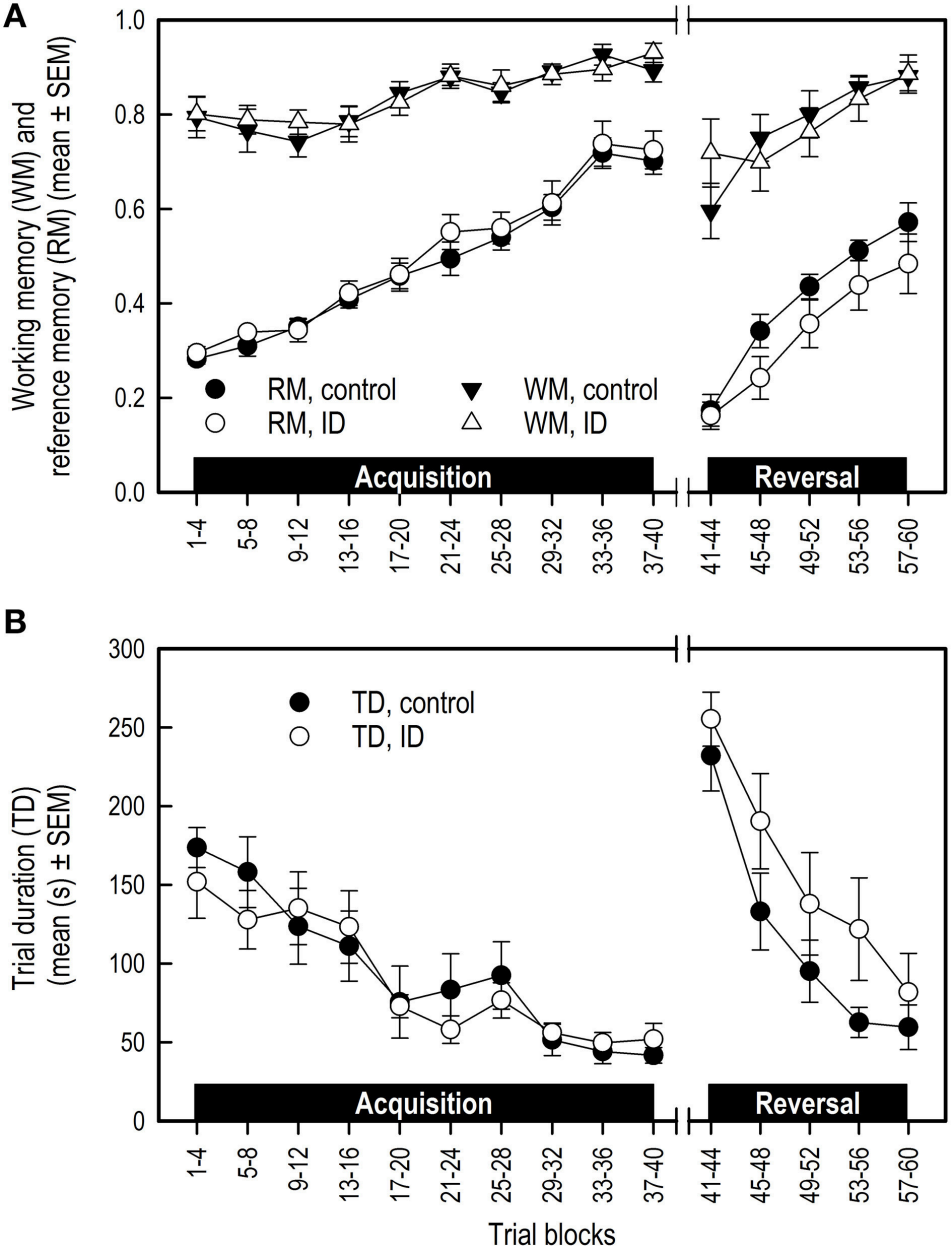
**Performance of ID and control piglets in the spatial cognitive holeboard task during the acquisition phase (trials 1–40) and the reversal phase (trials 41–60)**. **(A)** Working memory (WM) and reference memory (RM) performance; **(B)** Trial duration (TD). Note that TD was analyzed statistically after log_10_ transformation of the block means whereas the untransformed block means and SEMs are depicted here. For the results of the statistical analyses, see Table 3. Mean values and standard error of the mean (SEM) are depicted per treatment group.

WM and RM were not affected by treatment in any phase (Table 3B; Figure 3A). All piglets showed an increase in WM and RM performance in both the acquisition and reversal phase, and a decrease in these measures in the transition to a different set of baited holes.

#### Latency first visit and trial duration

**Figure 4:**
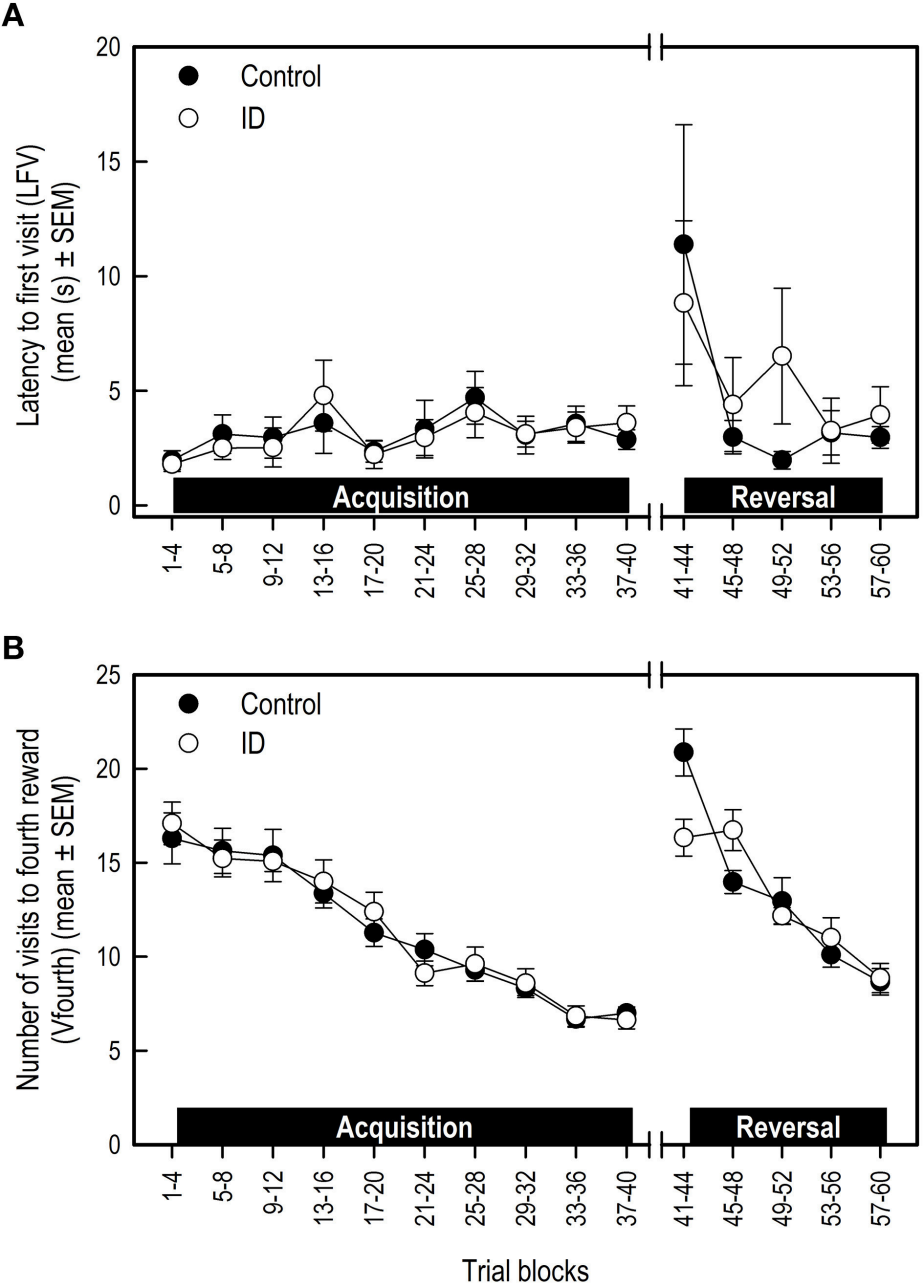
**Performance of ID and control piglets in the spatial cognitive holeboard task during the acquisition phase (trials 1–40) and the reversal phase (trials 41–60)**. **(A)** The latency to the first visit (LFV); **(B)** The number of hole visits until the fourth reward was found (Vfourth). For the results of the statistical analyses, see Table 3. Mean values and standard error of the mean (SEM) are depicted per treatment group.

There was no treatment effect on LFV in the acquisition and transition phase (Table 3B; Figure 4A). However, control animals showed a steeper decline in LFV in the reversal phase than ID animals (Treatment by Trial blocks interaction: *p* = 0.046).

Trial duration (TD) was not affected by treatment in any phase. TD decreased in all animals in the acquisition and reversal phase, and increased in the transition phase (Table 3B; Figure 3B).

#### Visits before fourth reward

All animals showed a decrease in the number of visits before the fourth reward was found (Vfourth) during the acquisition and reversal phase, and an increase in Vfourth in the transition phase (Table 3B; Figure 4B). There was no effect of treatment or Treatment by Trial blocks interaction on Vfourth in the acquisition phase.

In the transition to a different set of baited holes, control animals showed a larger increase in Vfourth than ID animals (Treatment by Trial blocks interaction: *p* = 0.02). This in turn may explain the observation that, in the reversal phase, control animals showed a steeper decrease in Vfourth than ID animals (Treatment by Trial blocks interaction: *p* = 0.002; Figure 4B).

## Discussion

The present study aimed to investigate whether piglets that remained with the sow until weaning, without providing them with additional feed or iron supplementation, could serve as a refined, less labor-intensive animal model for iron deficiency in humans. In this refined model, early maternal deprivation as a putative intervening factor was eliminated. Leaving the piglets with a sow until weaning also spares them the welfare-compromising stress of early removal from the sow.

Hematology, growth, and cognitive performance was assessed in ID and control piglets. Our results show that piglets reared by a sow without iron supplementation until weaning at 4 weeks of age did not become clinically anemic. Moreover, their long-term growth and cognitive performance was unaffected. In comparison with previous studies, early-life iron deficiency-induced clinical anemia seems essential in retarding growth and developing long-term impairments of memory performance.

### Blood iron values

Blood iron values were lower in ID animals than in control animals during treatment. After weaning and transition to iron-sufficient feed, blood iron values of ID piglets recovered to normal, comparable to those of control animals. This finding confirms that sow milk is low in iron content (Csapó et al., 1996).

#### No clinical anemia in ID animals

Surprisingly, pre-weaning hematocrit (Hct) and hemoglobin (Hb) values of ID animals did not decrease, and their serum iron values even rose between 1 and 3.5 weeks of age. In contrast, in our previous study assessing the effects of a more severe dietary ID treatment in young piglets (Antonides et al., 2015b), there was a steep decline in Hct, Hb, and serum iron during treatment in the ID animals. In that study, control animals received a 200 mg iron injection on day 3, ID animals received a saline injection. All piglets were separated from the sow at 4–6 days of age. Then, control animals were fed an iron sufficient diet (88 mg iron/kg diet) and ID animals an iron deficient diet (22 mg iron/kg) for 28 days. Similarly, piglets in the study by Rytych et al. (2012) were separated from the sow 48 h after farrowing and received a control (100 mg iron/kg), mildly (25 mg iron/kg) or severely (10 mg iron/kg) ID milk diet for 4 weeks. Similar to our previous study (Antonides et al., 2015b), the mildly and severely ID piglets in the study by Rytych et al. (2012) showed a decline in Hct, Hb, and serum iron values during treatment. In both studies, all ID animals (mild and severe) became clinically anemic, whereas the ID animals in the current study did not.

We argue that, comparing the results by Antonides et al. (2015b) and Rytych et al. (2012) with the findings of the present study, it is highly likely that piglets had access to an external source of iron while they were housed with the foster sows. A study by Sansom and Gleed (1981) investigated consumption of sow feces by suckling piglets by radioactive labeling the sows' feed and, consequently, their feces. They showed that piglets ingest around 20 g of sow feces per day (ranging from 5 to 85 g). This, they argue, may prevent piglets from becoming anemic, provided that one gram of fresh feces contains ~2 mg iron. Another possibility is that the piglets in our study consumed feed that was spilled by the foster sows. This could explain why, contrary to expectation, the blood iron values of our ID piglets did not decrease during treatment. We thus assume that these two external oral sources (feces and/or spilled feed) could have provided sufficient iron to prevent the development of anemia.

#### Control animals: iron administration and serum iron values

In two recent iron deficiency studies in piglets, control animals receiving an iron supplementation of 200 mg iron (Rytych et al., 2012; Antonides et al., 2015b) showed serum iron values at 4 weeks of age that were nearly twice as high as that of the control animals in the present study, which received two injections containing lower doses of iron. Additionally, the serum iron values of the control animals in Antonides et al. (2015b) were much higher during than after treatment (Rytych et al. did not measure after treatment). This may indicate that the amount of iron that was administered in those studies was indeed excessive, as argued by Lipiñski et al. (2010).

### Growth unaffected in ID animals

The most frequently used read-out parameter of performance and health of animals is growth (de Onis et al., 2006). In our study, withholding piglets from iron supplementation and additional feed before weaning did not affect their growth. Figure 2 suggests that control animals had slightly higher weights, yet this finding was not confirmed statistically. A study by Yu et al. (2002) also showed no effects of an iron injection on growth performance in piglets. In their study, by offering creep feed, piglets did not become clinically anemic. In our previous iron deficiency study in piglets, ID piglets did become clinically anemic and showed impaired growth (Antonides et al., 2015b). Our study results complement the findings of Yu et al. (2002) that early-life iron deficiency that does not lead to anemia does not affect growth.

### No effects of ID treatment on memory performance

As in previous studies using the holeboard task for pigs (Arts et al., 2009; Gieling et al., 2012; Antonides et al., 2015a,b), all piglets in our study acquired the holeboard task: their memory scores improved and trial durations declined during the course of training.

In the habituation trials, in which all 16 holes were baited, no differences were found for the total number of visits or total rewards found between treatment groups. In contrast, in our previous iron deficiency study inducing more severe dietary ID in young piglets leading to anemia (Antonides et al., 2015b), ID piglets made less total visits and found less rewards than control animals in the habituation trials. This indicates that cognitive deficits were already apparent in ID animals before formal training in the holeboard started in that study.

The most important indicators of memory performance in the holeboard task are WM and RM, which are forms of short- and long-term memory, respectively. We compared the RM scores during both the acquisition and the reversal phase of the control animals of the present study with those of the control animals of our previous iron deficiency study (Antonides et al., 2015b, see Supplementary Figure 1). Visual inspection of the RM learning curves corroborates the notion that the control groups of both studies showed a very similar RM performance. If maternal deprivation would have had an effect on the piglets' memory performance, then the learning curves of the pigs in these two studies would have been different. This supports the premise that maternal deprivation did not affect the performance of control pigs in the previous study. Nonetheless, we cannot completely exclude the possibility that the interaction of maternal deprivation and iron deficiency caused the cognitive deficits found in the ID pigs of our previous study. To test this possibility, the effects of iron deficiency must be compared between sow-reared and sow-deprived pigs in one and the same study. However, it will be challenging to match the degree of iron deficiency in such a study.

Visual inspection of Figure 3 suggests that the control group had higher RM scores and shorter TD in the reversal phase than the ID group. These impressions, however, were not confirmed statistically. We found that control pigs showed a steeper decline in LFV and in errors before finding the fourth reward (Vfourth) in the reversal phase than ID pigs. This is an indication that control animals learned faster in the reversal phase. However, LFV showed strong variation over the trial blocks, making it an unreliable measure on which to base conclusions.

Vfourth was higher in control animals at the start of the reversal phase, explaining the steeper decline in control animals during the reversal phase. The higher score for this measure in the first trial block of the reversal may indicate that control animals had a stronger recollection of their original set of baited holes than ID animals. This may be due to better memory. However, considering the lack of any effects on other memory scores, these findings are not a strong indicator of treatment effects on memory performance.

In our previous study (Antonides et al., 2015b), RM performance was impaired in ID piglets in both the acquisition and reversal phase. Comparably, piglets fed a severely ID diet for 4 weeks in the study by Rytych et al. (2012) were unable to acquire a T-maze task and piglets fed a mildly ID diet showed impaired reversal learning in the task compared to control animals.

In a review on iron deficiency studies in both humans and animals, McCann and Ames (2007) report that induced iron deficiency that does not lead to anemia in general does not lead to impaired growth or reduced performance in behavioral tasks. Our findings corroborate this notion in pigs. We consider the proposed refined and less invasive piglet model that we assessed in this study unsuited to serve as an animal model for iron deficiency in humans, as growth and memory of ID animals was unaffected.

However, the results of our study do indicate that the severity of the induced early-life iron deficiency crucially determines the impact on long-term development and cognition in piglets. The development of anemia seems a reliable indicator and predictor of long-term induced deficits caused by iron deficiency.

## Conclusion

The aim of the present study was to assess whether piglets left at the sow until weaning without iron supplementation or additional feed could serve as a refined, less labor-intensive piglet model for iron deficiency in humans. Pre-weaning hematocrit, hemoglobin and serum iron were lower in ID animals than in control animals. These values recovered to normal after weaning, when all piglets were fed the same iron-sufficient pig feed. Surprisingly, pre-weaning hemoglobin and hematocrit values did not decrease in ID animals during treatment, and their serum iron values even increased. Importantly, ID piglets did not become clinically anemic, as indicated by their hemoglobin values. This suggests that piglets had access to an external source of iron. As no additional feed was provided until weaning and sow milk is low in iron content, we argue that piglets probably consumed feces or spilled feed from the sow, preventing them from becoming anemic. Growth and memory performance in the holeboard task were unaffected in ID animals. Our results suggest that, as ID animals did not become clinically anemic, the imposed iron deficiency was not severe enough to cause long-term developmental or cognitive deficits. Our proposed animal model is thus not suited as a refined animal model for iron deficiency in humans. However, we did find that early-life iron deficiency that does not result in anemia does not have irreversible effects on long-term development and memory performance in piglets. Thus, the development of anemia in early-life iron deficiency seems to crucially determine whether long-term detrimental effects on physical and mental development arise.

## Author contributions

AA: Design of experiment, selection of animals, treatment of animals, statistical analyses, interpretation of results, writing of paper. SvL: Selection and treatment of animals, training and testing of the animals, interpretation of results, writing student report. FvdS: Design of experiment, feedback on experiment and reports, statistical analyses, interpretation of results. RN: Design of experiment, feedback on experiment and reports, interpretation of results.

### Conflict of interest statement

The authors declare that the research was conducted in the absence of any commercial or financial relationships that could be construed as a potential conflict of interest.

## Supplementary material

Supplementary Figure 1 **Comparison between reference memory scores of the control animals of our previous iron deficiency study (Antonides et al., 2015b), conducted in 2013, and of the control animals of the current study, conducted in 2015**.

Supplementary Table 1 **Overview of the composition of the experimental groups housed per pen after weaning at 4 weeks of age, and information of the piglets used in this study**.

Supplementary Table 2 **Number of samples per blood collection time point for the analysis of hemoglobin, hematocrit, and serum iron values**.
