# Supplementary figures and images for: Non-anemic Iron Deficiency from Birth to Weaning Does Not Impair Growth or Memory in Piglets
Source: Front Behav Neurosci. 2016 Jun 14;10:112. doi: 10.3389/fnbeh.2016.00112 (PMC4905972; doi:10.3389/fnbeh.2016.00112)

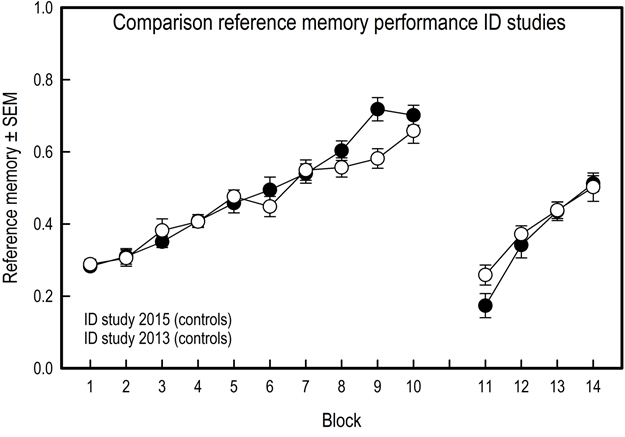

Supplement: Supplementary Figure 1 — Comparison between reference memory scores of the control animals of our previous iron deficiency study (Antonides et al., 2015b), conducted in 2013, and of the control animals of the current study, conducted in 2015. [file Image1.PNG]
