# Supplementary material for: Non-anemic Iron Deficiency from Birth to Weaning Does Not Impair Growth or Memory in Piglets
Source: Front Behav Neurosci. 2016 Jun 14;10:112. doi: 10.3389/fnbeh.2016.00112 (PMC4905972; doi:10.3389/fnbeh.2016.00112)
Supplement: Supplementary Table 1 — Overview of the composition of the experimental groups housed per pen after weaning at 4 weeks of age, and information of the piglets used in this study. [file Table1.DOCX]

**Supplementary Table 1.** Overview of the composition of the experimental groups housed per pen after weaning at 4 weeks of age, and information of the piglets used in this study. Siblings are depicted next to each other, one of them was housed in pen one, the other in pen two. Data marked in red show litter pairs that deviated from our protocol (according to which same sex siblings should be assigned to the two conditions and pens; also, of all piglets in the study, one piglet was not reared by a foster sow).

| ***Information of piglets used in this study*** | | | |  | |  | |  | |  | |  | |
| --- | --- | --- | --- | --- | --- | --- | --- | --- | --- | --- | --- | --- | --- |
|  | **Pen 1** | | | | | | **Pen 2** | | | | | | |
| **Litter** | **Pig ID** | **Treatment** | **Gender** | | **Foster Sow** | | **Pig ID** | | **Treatment** | | **Gender** | | **Foster Sow** |
| 1 | 1 | ID | **M** | | **3** | | 12 | | Control | | **F** | | **1** |
| 2 | 2 | ID | F | | **3** | | 13 | | Control | | F | | **1** |
| 3 | 3 | ID | M | | 1 | | 14 | | Control | | M | | 1 |
| 4 | 4 | Control | F | | 1 | | 15 | | ID | | F | | 1 |
| 5 | 5 | Control | F | | 1 | | 16 | | ID | | F | | 1 |
| 6 | 6 | ID | M | | 2 | | 17 | | Control | | M | | 2 |
| 7 | 7 | ID | M | | 2 | | 18 | | Control | | M | | 2 |
| 8 | 8 | Control | M | | 2 | | 19 | | ID | | M | | 2 |
| 9 | 9 | ID | F | | 2 | | 20 | | Control | | F | | 2 |
| 10 | 10 | Control | F | | ***** | | 21 | | ID | | F | | **3** |
| 11 | 11 | Control | **F** | | 3 | | 22 | | ID | | **M** | | 3 |

*: This piglet was not housed with a foster sow, but did receive iron injections according to the adjusted schema used in this study.
