# Supplementary material for: Non-anemic Iron Deficiency from Birth to Weaning Does Not Impair Growth or Memory in Piglets
Source: Front Behav Neurosci. 2016 Jun 14;10:112. doi: 10.3389/fnbeh.2016.00112 (PMC4905972; doi:10.3389/fnbeh.2016.00112)
Supplement: Supplementary Table 2 — Number of samples per blood collection time point for the analysis of hemoglobin, hematocrit, and serum iron values. [file Table2.DOCX]

**Supplementary Table 2.** Number of samples per blood collection time point for the analysis of hemoglobin, hematocrit and serum iron values.

| **Number of samples per time point of blood collection** | | | | | |
| --- | --- | --- | --- | --- | --- |
| **Measure** | **Treatment** | **Age in weeks** | | | |
|  |  | **1** | **3.5** | **6** | **12** |
| **Hemoglobin (Hb)** | ID | 10 | 11 | 11 | 8 |
|  | control | 10 | 11 | 11 | 10 |
| **Hematocrit (Hct)** | ID | 10 | 11 | 11 | 8 |
|  | control | 10 | 11 | 11 | 10 |
| **Serum Fe** | ID | 10 | 11 | 11 | 11 |
|  | control | 8 | 11 | 11 | 11 |
